# A new design for an artificial cell: polymer microcapsules with addressable inner compartments that can harbor biomolecules, colloids or microbial species†
†Electronic supplementary information (ESI) available: Additional figures showing the microfluidic device used in this study as well as the sorting of MCCs with magnetic inner compartments. See DOI: 10.1039/c7sc01335c


**DOI:** 10.1039/c7sc01335c

**Published:** 2017-08-17

**Authors:** Annie Xi Lu, Hyuntaek Oh, Jessica L. Terrell, William E. Bentley, Srinivasa R. Raghavan

**Affiliations:** a Department of Chemical and Biomolecular Engineering , University of Maryland , College Park , MD 20742 , USA . Email: sraghava@umd.edu; b Fischell Department of Bioengineering , University of Maryland , College Park , MD 20742 , USA

## Abstract

Eukaryotic cells have an architecture consisting of multiple inner compartments (organelles) such as the nucleus, mitochondria, and lysosomes. Each organelle is surrounded by a distinct membrane and has unique internal contents; consequently, each organelle has a distinct function within the cell. In this study, we create biopolymer microcapsules having a compartmentalized architecture as in eukaryotic cells. To make these capsules, we present a biocompatible method that solely uses aqueous media (*i.e.*, avoids the use of oil phases), requires no sacrificial templates, and employs a minimal number of steps. Our approach exploits the electrostatic complexation of oppositely charged polymers dissolved in aqueous media. Specifically, droplets of an anionic biopolymer are generated using a simple microcapillary device, with the droplets being sheared off the capillary tip by pulses of gas (air or nitrogen). The liquid droplets are then introduced into a reservoir whereupon they encounter multivalent cations as well as a cationic biopolymer; thereby, a solid shell is formed around each droplet by electrostatic interactions between the polymers while the core is ionically cross-linked into a gel. In the next step, a discrete number of these capsules are encapsulated within a larger outer capsule by repeating the same process with a wider capillary. Our approach allows us to control the overall diameter of these multicompartment capsules (MCCs) (∼300–500 μm), the diameters of the inner compartments (∼100–300 μm), and the number of inner compartments in an MCC (1 to >5). More importantly, we can encapsulate different payloads in each of the inner compartments, including colloidal particles, enzymes, and microbial cells, in all cases preserving their native functions. A hallmark of biological cells is the existence of cascade processes, where products created in one organelle are transported and used in another. As an initial demonstration of the capabilities afforded by our MCCs, we study a simple cascade process involving two strains of bacteria (*E. coli*), which communicate through small molecules known as autoinducers. In one compartment of the MCC, we cultivate *E. coli* that produces autoinducer 2 (AI-2) in the presence of growth media. The AI-2 then diffuses into an adjacent compartment within the MCC wherein a reporter strain of *E. coli* is cultivated. The reporter *E. coli* imbibes the AI-2 and in turn, produces a fluorescence response. Thus, the action (AI-2 production) and response (fluorescence signal) are localized within different compartments in the same MCC. We believe this study is an important advance in the path towards an artificial cell.

## Abstract 2

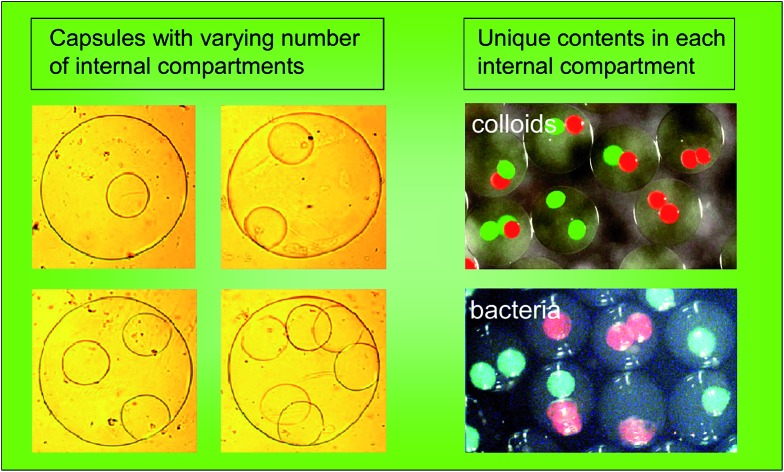
Multicompartment capsules with control over the contents of each inner compartment are prepared by a simple, oil-free technique.

## Introduction

**Fig. 1:**
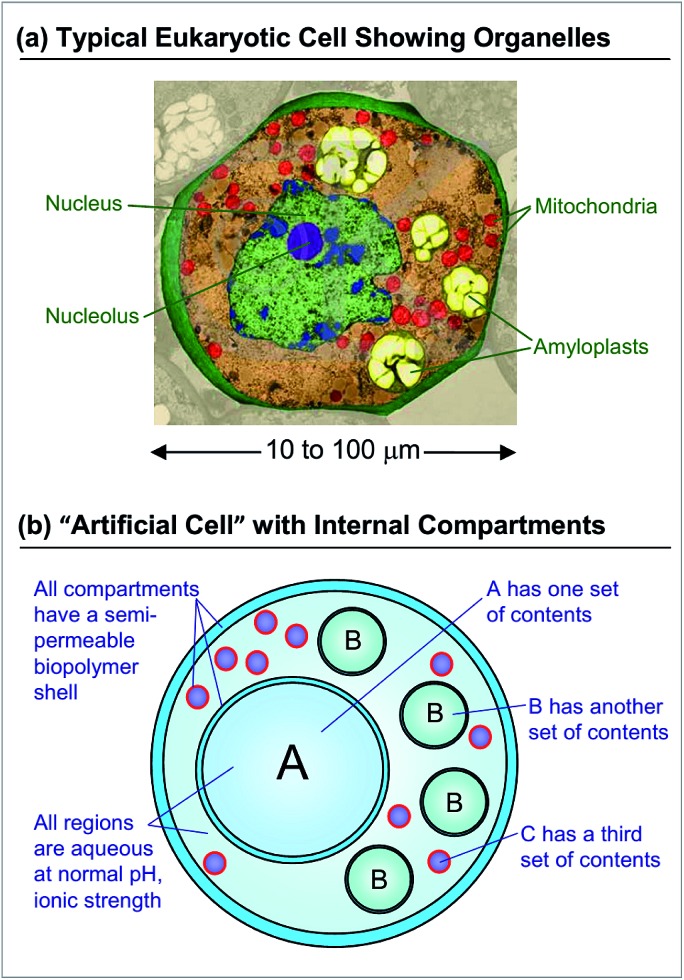
Architecture of a typical eukaryotic cell and of a cell-mimicking microcapsule. (a) Cross-section of a parenchymal cell from a lily plant with false-color rendering to indicate the different organelles (©SCIENCEphotoLIBRARY, used with permission). (b) Schematic of a biopolymer-based microcapsule that mimics the structure in (a). Three different types of internal compartments (A, B, C) are shown, with each type having a similar size and similar contents. The entire system is based on water at physiological pH and ionic strength. We refer to this type of structure as a multicompartment capsule (MCC).

Over the past two decades, the search for new materials has increasingly drawn inspiration from biology.^1^,^2^ Although numerous advances in biomimetic materials have now been reported, there still remains a large gap between structures that can be designed in the laboratory and those found in biology. A prototypical example is that of a single (eukaryotic) cell, shown in cross-section in Fig. 1a.^3^ The cell is a remarkable multifunctional material. It is capable of synthesizing proteins and lipids, storing and harvesting energy, storing and retrieving genetic information, and recycling used or defective material.^3^ The ability of the cell to accomplish these diverse tasks is intimately related to its *architecture*, *i.e.*, to the fact that it has distinct *internal compartments* (organelles), each bounded by a lipid membrane. Each type of organelle has a different function: for example, in animal cells, the Golgi bodies serve as centers for protein and lipid synthesis, the mitochondria as the “power plants” where energy is stored, and the lysosomes as the “recycling centers” where proteins are degraded.^3^ The function of each organelle is tied to its unique internal constituents; at the same time, the membrane around the organelle tightly regulates the entry and exit of molecules. For example, lysosomes maintain a highly acidic pH, which enables hydrolytic degradation of proteins—however, this acid does not pass through into the surrounding cytoplasm.

In recent years, several researchers have attempted to create artificial cells (also called “protocells”) with the same kind of compartmentalized architecture.^4^–^11^ While these studies have produced many impressive results,^12^–^29^ a simple and versatile method to create multicompartment protocells is still lacking. In this context, it is useful to list the features that such structures should ideally possess. Consider the schematic in Fig. 1b of a cell-mimic that resembles the cell shown in Fig. 1a. This is overall envisioned to be a large container or capsule with several smaller compartments inside it. To create such a structure, one has to control the number of internal compartments, their sizes, and the contents inside each of them. For example, in the schematic, a total of 15 inner compartments are shown: a large A compartment with certain contents, four smaller B compartments with another set of contents, and ten much smaller C compartments with a third set of contents. It is also important that all these compartments have an aqueous interior with a composition (pH and ionic strength) compatible with biological media. That is, it should be possible to encapsulate payloads such as biomolecules (proteins, nucleic acids) and even live cells (microorganisms, mammalian, plant cells) in these compartments. Finally, to facilitate adoption by a variety of researchers, it would be helpful if the entire structure could be made using inexpensive starting materials and using a simple, quick, and straightforward process.

With regard to the above list of requirements, current attempts at creating multicompartment protocells have some drawbacks. In some cases, the compartments may lack a membrane,^9^ or may have coexisting oil and water phases,^28^ or may be stable only in non-aqueous solvents.^29^ The oil or solvents can be problematic for encapsulation of biological payloads and moreover, a real cell is not an emulsion in terms of its structure. In other cases, the method to synthesize individual compartments involves layer-by-layer assembly of polymers around a core template, followed by removal of the template.^18^,^19^ Layer-by-layer assembly is a laborious process involving 50 or more consecutive steps. Besides, the necessity for a template adds further complexity since conditions for subsequent removal of a template tend to be harsh (*e.g.*, dissolution of silica using acids). Moreover, when the template is removed, one obtains a core that does not contain any payload; strategies then have to be devised to load the empty core with appropriate contents. Some of the best examples of protocells are polymersome-in-polymersome^21^–^27^ and liposome-in-liposome^12^–^16^ structures, which have recently been used to run enzymatic cascade reactions.^21^,^26^,^27^ However, the techniques to make these structures are rather complex, and it is difficult to control the number of individual compartments as well as their specific contents. Moreover, polymersomes require block copolymers that typically need to be synthesized and are not commercially available.

In this study, we present a new approach toward a rudimentary artificial cell based on polymer microcapsules. The term ‘capsule’ refers to structures with an inner aqueous core surrounded by a polymeric shell that is permeable to small molecules and ions, but not to macromolecules or nanoparticles.^4^ We refer to our overall structure as a “multicompartment capsule” (MCC), *i.e.*, a capsule with multiple smaller compartments in it, as depicted in Fig. 1b. All capsules are made here by electrostatic complexation^30^–^36^ using common biopolymers such as alginate^37^ and chitosan^38^ that are widely used in biomedical studies. Biopolymer-bearing aqueous droplets are generated by a simple microfluidic device built from glass or plastic tubing and using pulses of gas (air); the droplets are subsequently converted to capsules by electrostatic complexation. A subsequent microfluidic step is used to encapsulate small capsules in a larger capsule. In the entire process, no immiscible oil phase is used, which means that we can readily encapsulate intact biological payloads such as proteins and cells in individual compartments of the MCC. Importantly, payload encapsulation and capsule formation are accomplished in a single step, and thus, we can precisely control the contents of each compartment in our MCC. Also, due to the use of inexpensive biopolymers and tubing, our method is readily accessible to any laboratory, and the same platform can be used to make a variety of cell-like structures. No access to microfabrication facilities or a clean room is necessary, and the device can be operated by anyone with minimal training.

As noted earlier, the cell-like structure of MCCs allows encapsulated payloads to be kept separate in distinct compartments, while the proximity of the compartments enables cascade reactions. Towards this end, we demonstrate that we can cultivate two strains of genetically engineered *E. coli* in adjacent compartments of an MCC. One *E. coli* strain is a producer (P),^39^–^41^ *i.e.*, it produces a small molecule called autoinducer-2 (AI-2) that is involved in a bacterial signalling process called quorum sensing (QS).^42^,^43^ The AI-2 formed in the producer compartment then diffuses into adjacent compartment(s) where a second reporter (R) strain of *E. coli* is cultivated. The reporter *E. coli* responds to the AI-2 by turning on a gene that produces a fluorescent protein.^40^,^41^ This allows the response in the reporter compartment(s) to be observed visually by fluorescence microscopy. Our experiments illustrate how the MCCs can be used to study a simple cascade process involving two microorganisms in close proximity within the same environment. In the future, the MCCs will allow us to juxtapose different types of microorganisms, including ones that normally cannot be cultured together. One could also explore cross kingdom communication^42^ or the co-culture of competitive species. Other applications for these MCCs are envisioned to arise in biomolecular catalysis, drug delivery, and tissue engineering.

## Results and discussion

### Preparation of individual compartments

**Fig. 2:**
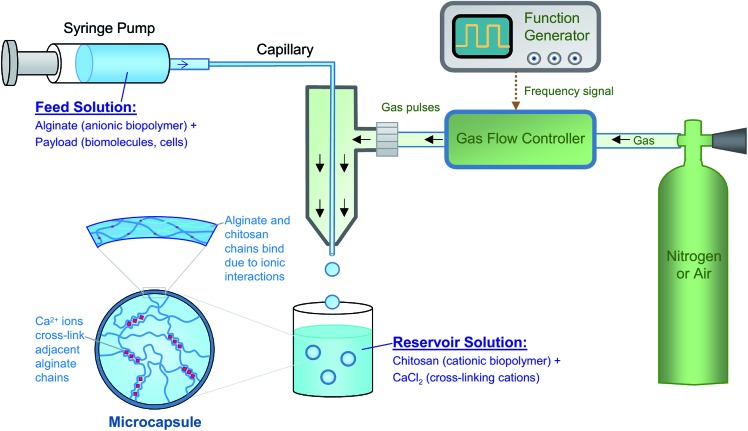
Synthesis of microcapsules by a water–gas microfluidic technique. Microdroplets bearing the anionic biopolymer, alginate as well as payloads of interest are generated by flowing the aqueous solution through a capillary. Pulses of gas (nitrogen or air) are sent through the annular region around the capillary. The frequency of the pulses is controlled by the function generator. Uniform aqueous droplets emerge from the tip of the capillary, and these are introduced into an aqueous reservoir solution containing the cationic biopolymer chitosan as well as the salt of a divalent cation (Ca^2+^). The droplets are thereby converted into microcapsules, with the shell being formed by electrostatic complexation between the anionic alginate and the cationic chitosan while the core is further strengthened by the Ca^2+^-induced cross-linking of alginate chains.

We first prepared individual microscale capsules using a water–gas microfluidic setup (Fig. 2). These capsules serve as the internal compartments in our MCC structure. Typical setups for droplet microfluidics use immiscible aqueous and oily phases, which are brought into contact at a T-junction or within a co-flow geometry.^44^,^45^ Our group^35^,^46^ and others^47^ have instead pioneered oil-free droplet microfluidics, where instead of the oil (which could be harmful to biological systems), an inert gas (either air or nitrogen) is used. The use of this method to form polymer capsules has been briefly mentioned elsewhere,^35^ but is described in more detail here. Our droplet generator consists of an inner glass capillary of inner diameter (ID) ∼50 μm, which is threaded through the end of a pipette tip (photo in Fig. S1†). The aqueous solution of interest is passed through this capillary, with the flow rate being controlled by a syringe pump. In the annular space surrounding the capillary, pulses of gas are dispatched by a function generator connected to a gas flow-regulator (Fig. 2). The pulses are applied over a very short duration (0.1 s) while the duration between consecutive pulses is controlled by the pulsing frequency *f* (see Fig. S1†).^48^ The gas flows as a sheath around the tip of the inner capillary, and for every pulse of gas, an aqueous droplet is dislodged from the capillary tip. The use of a function generator is a key innovation in our approach; as will be shown, it enables precise control over the size of droplets.

Aqueous droplets are converted into capsules by contact with the reservoir solution (Fig. 2). Several chemistries can be used in this context, but the focus here is on the biopolymer sodium alginate. Alginate is an anionic polysaccharide that is compatible with biomolecules as well as biological cells.^37^ It is well-known that alginate solutions can be converted to gels by addition of multivalent cations like Ca^2+^ or Sr^2+^; these cations form cross-linking zones called “egg-box” junctions between adjacent alginate chains.^37^ In our setup, we use 2.25 wt% alginate in the droplet generator, while the reservoir contains 1 wt% of CaCl_2_ and 1 wt% of chitosan. Chitosan is a cationic polysaccharide^38^ and the one used here has a low molecular weight of ∼5000 Da, *i.e.*, it is an oligomer and is soluble at neutral pH.^30^ When the alginate-bearing droplets contact the reservoir solution, two processes occur. The anionic alginate and the cationic chitosan undergo electrostatic complexation,^30^,^35^ where the oppositely charged polymers bind together and form a gel. This process begins at the surface of the droplet, forming a shell around the droplet (Fig. 2), and proceeds inward. At the same time, the Ca^2+^ ions in the solution also diffuse into the droplet and cross-link the alginate chains. The combination of the two processes results in the conversion of droplets into stable capsules. We use the term ‘capsule’ to denote the fact that the shell generally has distinct properties from the core.^31^–^35^ Note that the Ca^2+^ ions will tend to diffuse all the way through the droplet, resulting in the entire core becoming a gel. The chitosan, being a macromolecule, will diffuse a shorter distance and will thus be confined near the shell. After a certain incubation time in the reservoir (typically about 30 min), the capsules are washed with phosphate-buffered saline (PBS) and then resuspended in PBS.

**Fig. 3:**
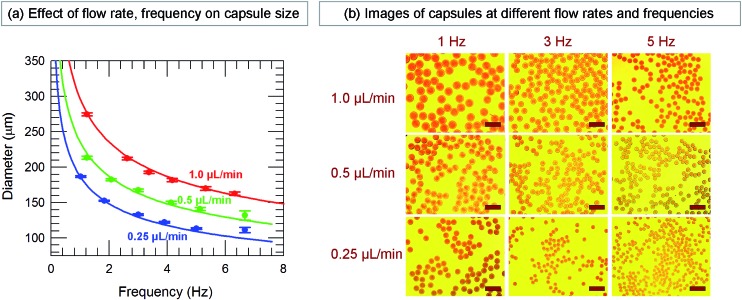
Effect of liquid flow rate (*Q*) and gas pulse frequency (*f*) on the size of microcapsules. (a) Plot of capsule diameter *vs.* frequency at three different flow rates. The values plotted are the means determined from image analysis and the error bars represent standard deviations about the mean. Up to *f* ∼ 6 Hz, the capsules are very uniform, with the standard deviations being <3%. The lines through the data are fits to eqn (1). (b) Optical micrographs of typical capsules generated at different *Q* (0.25, 0.5, 1.0 μL min^–1^) and *f* (1, 3, 5 Hz). Scale bars in the images are 500 μm.

In our approach, since each droplet is converted into a capsule, the size of the droplets dictates the size of the capsules. The variables that affect droplet size are the feed (liquid) flow rate *Q*, which is controlled by the syringe pump, and the pulsing frequency *f* of the gas, which is controlled by the function generator and is varied between 1 to 7 Hz in our experiments. The effects of these two variables on capsule size are shown in Fig. 3. The capsule diameter is plotted against frequency in Fig. 3a for three different liquid flow rates. Optical micrographs of capsules obtained at specific conditions are shown in Fig. 3b. In all cases, the capsules are very uniform, with the polydispersities in their diameter being <3%. Fig. 3a shows that capsule size can be decreased by either lowering the liquid flow rate *Q* or increasing the pulsing frequency *f*. These trends can be understood based on how *Q* and *f* affect the droplet volume. Assuming that every pulse of gas results in exactly one droplet (and hence one capsule), we can express the droplet volume as *V*_droplet_ = *Q*/*f*. The capsule is slightly smaller than the droplet due to shrinking, and we empirically put *V*_capsule_ = *a*(*V*_droplet_) with *a* ≤ 1. In turn, the capsule diameter *d*_cap_ can be calculated as:1
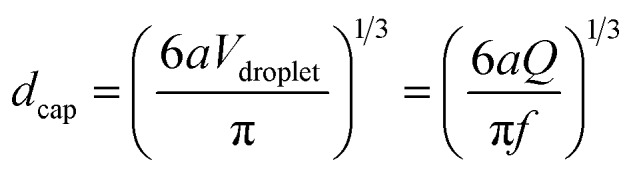



The lines in Fig. 3a are fits of eqn (1) at each flow rate *Q* with a value of *a* = 0.81 for all three cases. An excellent match is seen between the predicted and measured capsule sizes for frequencies ranging from 1 to 6 Hz. Above 6 Hz, the discrepancy between the two sizes is likely because droplets are no longer generated at the rate of one per pulse of gas. Fig. 3a can be used to pre-determine the conditions (*Q*, *f*) needed to obtain capsules of any specific diameter between about 100 to 300 μm. This is the advantage provided by the function generator; without it, one does not have the same control over capsule size.^48^ Note that the data in Fig. 3a were collected at a particular diameter of the capillary and at a specific gas pressure (14 psi). The pressure is set by the gas flow-regulator, and its value was chosen such that it was high enough to dislodge the liquid droplet, but not too high as to break the droplet into smaller units. Once this pressure is set, the capsule size is controlled by eqn (1) regardless of the fluid properties.

### Preparation of MCCs

**Fig. 4:**
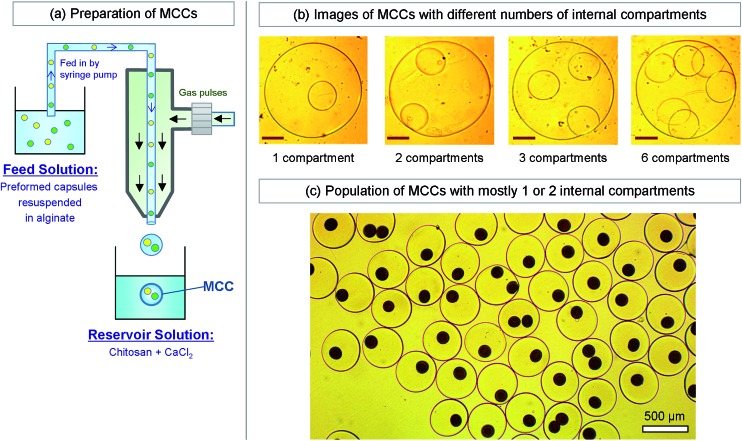
Preparation and typical images of multicompartment capsules (MCCs). (a) Preparation of MCCs by the same water–gas microfluidic method described in Fig. 2. A suspension of preformed capsules in an alginate solution is used as the liquid feed through the capillary. Gas pulses are used to dislodge uniform droplets from the tip of the capillary, and the droplets are then introduced into the reservoir solution containing chitosan and Ca^2+^. The droplets are thereby converted into MCCs. (b) Optical micrographs of individual MCCs with different numbers of (identical) internal compartments. The scale bars in the images are 100 μm. (c) Optical micrographs of a population of MCCs having either one or two (identical) internal compartments. The compartments all have a brown color because they contain magnetic Fe_3_O_4_ nanoparticles.

The second part of our approach is the formation of MCCs with the capsules from the first step as inner compartments (Fig. 4). For this, we start with the capsules from the first step suspended in PBS and add 2 wt% alginate. We then use this capsule dispersion as the liquid feed into our gas–liquid droplet generator (Fig. 4a). The setup is identical to the one in Fig. 2, with the same function generator, gas flow-regulator, and syringe pump. The only difference is that we increase the diameter of the inner glass capillary to 200 μm so as to accommodate the suspended capsules. The reservoir composition is also identical to that in Fig. 2. Using this procedure, we are able to form MCCs, and these can again be washed and resuspended in PBS. For the simplest case of identical inner compartments, the key variable is the number of such compartments. Fig. 4b shows optical micrographs of MCCs with one, two three, and six inner compartments. The diameter of the MCCs is ∼400 μm while the diameter of each inner compartment is about 100 μm. Thus, we are able to readily form MCCs with different internal architecture using our method. Note that the lumen of the MCCs surrounding the compartments will also be an alginate gel, similar to the lumen of each compartment.

How to sort or isolate MCCs with a particular number of internal compartments? As mentioned above, in forming the MCCs, we use a dispersion of capsules in alginate solution as the feed to our droplet generator. The higher the concentration (number density) of capsules in solution, the greater the average number of compartments in a given droplet (and hence in the subsequent MCC). However, droplet generation is a stochastic process, and therefore there will be many variants. For example, Fig. 4c shows a population of MCCs that exhibit one or two internal compartments, obtained by using a moderate concentration of capsules in the feed. The dark brown color of the compartments is due to the presence of magnetic Fe_3_O_4_ nanoparticles (MNPs, 10 nm diameter) in each of them. To separate a particular kind of MCCs from the rest, we can conveniently exploit their relatively large size, *i.e.*, the fact that they are large enough to be seen and manipulated individually using an optical microscope. Thus, for example, MCCs with exactly two internal capsules can be sorted manually from the above population using a pipette tip on a microscope slide. While this method is rudimentary, it is effective at the length scale studied here.

When the population of MCCs is very large or if their sizes are much smaller, manual sorting is not convenient. In this case, we mention two alternative approaches for sorting. First, we can exploit the fact that MCCs with different numbers of internal compartments are likely to have different densities. Sorting can then be done using a centrifuge. The density differences can be accentuated by loading nanoparticles with a higher density, such as MNPs, in the core of each inner compartment; in that case, the number of compartments will dictate the overall density of each MCC. A second related approach is to exploit a magnetic field in the case of MNP-bearing compartments. For example, Fig. S2† shows that we can use an external bar magnet to isolate MCCs with one or two such compartments while leaving behind the capsules with no inner compartments. Thereafter, a magnetic or density-based approach can be used to further separate the 2-compartment MCCs from the 1-compartment ones.

### MCCs with distinct compartments

The utility of MCCs can be truly exploited only if we have the ability to place distinct contents in every compartment. Our synthesis method conveniently provides this ability. In forming the original capsules, which serve as the inner compartments, any payload that is included along with the feed solution of alginate gets sequestered in the core of the capsules. The alginate solution is typically a thin, aqueous fluid at neutral pH, and its ionic strength can also be adjusted to physiological levels (150 mM). Thus, this solution is compatible with all kinds of biological payloads, including proteins, nucleic acids, micro-organisms, and mammalian cells. Based on previous studies with similar biopolymer capsules, both in our lab^32^–^36^ and elsewhere,^31^,^38^ it is known that the shell of these capsules allows small molecules and ions to pass through, but acts as a barrier to any species that are at the nanoscale or larger. For example, we have encapsulated enzymes or fusion proteins with molecular weights of 80 kDa and higher (*i.e.*, a radius of gyration *R*_g_ ∼10–30 nm),^33^–^35^ inorganic nanoparticles with sizes of a few nm, and liposomes or vesicles with sizes of ∼100 nm.^32^,^33^ Such nanoscale entities remain entrapped in the capsule lumen and do not escape through the shell into the external medium. Also, it is easy to mix and match any or all of these payloads in the capsules.

**Fig. 5:**
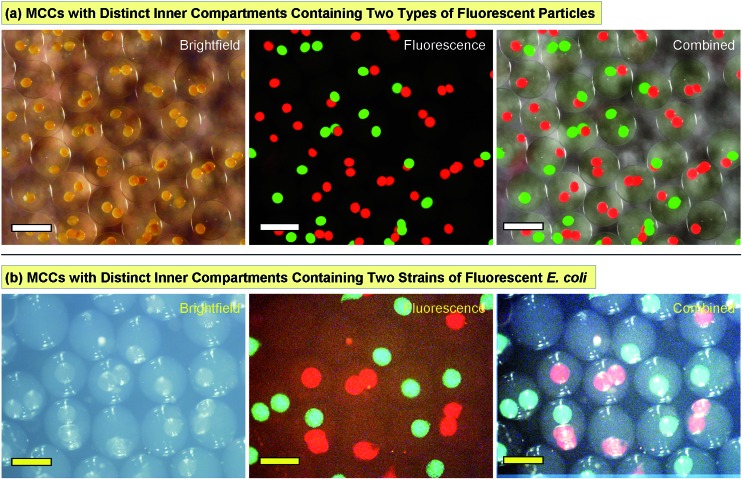
Multicomparment capsules (MCCs) with internal compartments bearing distinct payloads. (a) The compartments have either green- or red-fluorescent colloidal particles (800 nm diameter). (b) The compartments have two strains of *E. coli* that express either green fluorescent protein (GFP) or red fluorescent protein (RFP). In both cases, the first two images are shown in brightfield and fluorescence mode, and the two are combined in the third image. Scale bars in (a) are 500 μm and in (b) are 1000 μm.

To demonstrate multiple compartments with distinct payloads in an MCC, we first employed two kinds of fluorescent colloids, exhibiting green and red fluorescence, respectively. Both particles had diameters of ∼800 nm. We used our microfluidic technique (Fig. 2) to produce one set of capsules with the green fluorescent particles in them. Then, we similarly produced another batch of capsules with the red fluorescent particles in them. The two batches of capsules were then combined in an alginate solution and this was used as the feed to produce MCCs, as per Fig. 4. The resulting MCCs are shown unsorted in Fig. 5a. Optical micrographs are shown in brightfield, fluorescence, and combined mode. We observe that most MCCs have one or two inner capsules (compartments), which is the result of using a moderate concentration of red and green capsules in the feed. All combinations are seen in the image, *i.e.*, MCCs with two red, two green, one red and one green, only one red, and only one green compartment. A few MCCs with more than two compartments are also seen. Note that in all cases, there is no leakage of fluorescence from the compartments to the lumen of the MCC or to an adjacent compartment, indicating that the particles remain localized within their respective compartments.

Next, we performed MCC synthesis with two distinct strains of bacteria (*E. coli*). Both strains were genetically engineered to detect a signaling molecule called autoinducer 2 (AI-2) and to respond by activating the genes for specific fluorescent proteins.^39^–^41^ One strain was engineered to produce green fluorescent protein (GFP) while the other to produce red fluorescent protein (RFP). AI-2 is a molecule that is synthesized by bacteria and involved in bacterial quorum sensing (see below). But in this first experiment, we add synthetic AI-2 to the solution and simply use it as a trigger to turn on bacterial responses (the bacteria in this case were mutants that could not synthesize their own AI-2).^39^–^41^ We again began by making capsules containing each strain of *E. coli*, then combining the two sets of capsules to produce MCCs. These MCCs are shown in Fig. 5b with the bacteria localized in distinct internal compartments. In the presence of growth (LB) medium and when placed on a shaker at 37 °C, the bacteria grow and form colonies inside their compartments. Moreover, when AI-2 is added to the medium, the bacteria are induced to fluoresce. Fig. 5b shows MCCs with combinations of red- and green-fluorescent compartments much like the MCCs with particles in Fig. 5a. Note that the bacteria are confined to their specific compartment and do not come into contact (these images were taken 1–2 h after the AI-2 was added). Thus, the MCCs permit simultaneous co-culture of two bacterial strains in their specific microenvironments.

### MCCs used to conduct a bacterial cascade process

Fig. 5b shows that we can successfully encapsulate and cultivate biological cells in their own compartments within MCCs. The next step is to attempt a cascade process involving such cells where a change occurring in one compartment of the MCC is transduced into a response in an adjacent compartment. For this, we use two genetically engineered *E. coli* strains that can participate in quorum sensing (QS). QS is an important process in bacterial communication where the behavior (phenotype) of a bacterial population changes when a minimum cell density (quorum) is reached.^42^,^43^ Changes in phenotype caused by QS include the expression of virulence factors or the formation of bacterial biofilms. Bacteria produce and release signaling molecules such as AI-2 that regulate QS. The ability of capsules and liposomes to interfere with bacterial QS pathways has been a topic of continued interest.^34^,^49^ For the experiments here, we have chosen two bacterial strains. One strain, *E. coli* BL21, is an AI-2 producer and compartments in the MCC with this strain are labeled P. The other strain, *E. coli* W3110, is an AI-2 reporter and its compartments are labeled R. The reporter *E. coli* are mutants that cannot synthesize their own AI-2, but create a green-yellow fluorescent protein called VENUS in response to AI-2.^33^,^34^ We made MCCs with combinations of R and P compartments in them. For easy identification, the R compartments were deliberately synthesized at a slightly larger size than the P compartments.

**Fig. 6:**
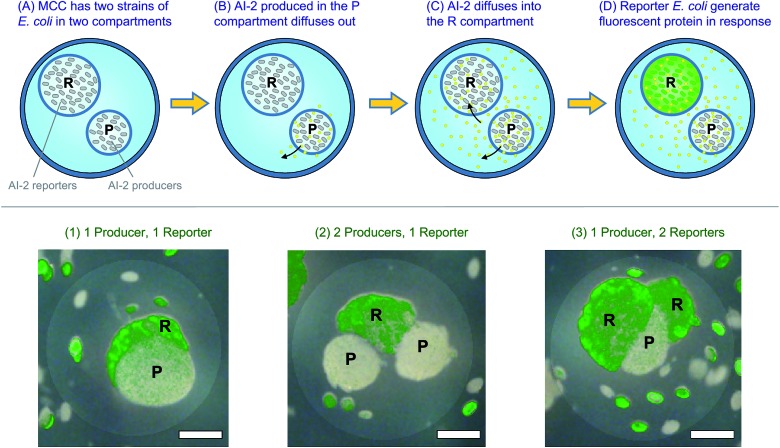
Demonstration of a bacterial cascade process using MCCs. (Top) The schematics show the sequence of events. (A) Initially, an MCC is studied in which two strains of *E. coli* are encapsulated in two distinct compartments. The strain in the P compartment is an AI-2 producer while the strain in the R compartment is an AI-2 reporter. Neither compartment is fluorescent at this stage. (B) As the bacteria grow, AI-2 (yellow dots) is synthesized in the P compartment. The AI-2 diffuses out into the capsule lumen. (C) The AI-2 then diffuses into the R compartment, where it turns on the reporter bacteria. (D) In turn, the reporter *E. coli* synthesize the fluorescent protein VENUS, and thus the entire compartment appears green under a fluorescence microscope. (Bottom) Combined brightfield + fluorescence microscopy images are shown for individual MCCs with different arrangements of P and R compartments. In (1), (2), and (3), there are at least one P and one R compartments in the MCC (note that the P compartments are deliberately made to be slightly smaller than the R ones). As expected, the images show fluorescence in the R compartment but not the P ones. Scale bars in the images are 250 μm.

The experiment over the course of time is schematically depicted in the top panel of Fig. 6. At time *t* = 0, the MCCs are placed in growth media at 37 °C (Panel A). At this stage, neither compartment of the MCC shows fluorescence. As time progresses (*t* = 4 to 6 hours), the cells grow and form small microcolonies in the compartments. AI-2 is produced in the P compartments and it diffuses out of these into the MCC lumen, or “cytoplasm” and from there into the R compartments (Panels B, C). Note that AI-2 is a small molecule with a molecular weight of 193 Da and thus can readily pass through capsule shells. When a sufficient concentration of AI-2 is reached in the R compartments (*t* > 12 h), the reporter *E. coli* respond by creating the fluorescent VENUS protein (Panel D). Thus, a fluorescence signal is expected in the R compartments (but not the P ones) after an induction time. This is exactly what we observe by fluorescence microscopy. The micrographs in the bottom panel of Fig. 6 are superpositions of fluorescence and brightfield images, and are taken after 24 h of culture. Incidentally, the fluorescence signal from VENUS shows up as a green color due to the filter settings on our microscope. The images are of single capsules with different combinations of P and R compartments. Image 1 has one P and one R (the R is behind the P and hence mostly obscured), image 2 has two P and one R, while image 3 has two R and one P. In all these cases, we see bright fluorescence in the R compartment(s) but not in the P ones.

Interestingly, in 24 h, we find that the bacteria have rapidly proliferated such that some of their microcolonies can no longer be contained within their home compartment. As a result, we see some colonies in the lumen of the MCC. Similar findings of microbes outgrowing their initial confines and leaking out into the external medium have been reported for the case of alginate capsules encapsulating yeast.^50^ In our case, the presence of the distally spaced microcolonies in the lumen demonstrate the distances by which AI-2 travels by diffusion over the period of observation. Overall, Fig. 6 demonstrates that bacteria remain viable owing to their ability to abstract energy and nutrients from the surrounding medium; more importantly, they continue to be capable of making and transducing signaling molecules. This demonstration illustrates that small molecules can be transmitted and received by viable cells contained in each compartment, revealing molecular “crosstalk” between the adjacent compartments.

## Conclusions

We have demonstrated a simple, scalable way to create MCCs. Our method addresses many of the issues with previous approaches. We use common, inexpensive biopolymers (alginate, chitosan) as precursors; these are biocompatible and widely used in biochemical and cellular studies. A water–gas microfluidic technique is developed to generate biopolymer-containing aqueous droplets, which are then converted to capsules upon contact with a reservoir solution. No immiscible phase (oil) is used in the entire process, which greatly simplifies isolation, cleanup and purification of the capsules. To form the capsules, we exploit the electrostatic complexation of oppositely charged biopolymers, along with ionic cross-linking. These processes are mild and do not involve any covalent bond formation; thus, they are biologically benign and compatible with labile payloads such as enzymes and microbial or eukaryotic cells. The above capsules are then combined in a second step using the same microfluidic setup to produce MCCs. Our approach provides control over the number and size of the inner compartments in an MCC and, most importantly, over the contents of each compartment. Compartments with enzymes, colloidal particles, and biological cells, can be juxtaposed within a given MCC.

A specific demonstration with MCCs in this study is of a cascade process between strains of *E. coli* in separate compartments. AI-2 generated by a producer strain of *E. coli* in one compartment diffuses over to the neighboring one(s), where a reporter strain of *E. coli* generates a fluorescent response. This experiment firstly shows that bacteria can be cultured in individual compartments of an MCC, just like in a Petri dish. For bacteria to thrive and grow, it is important that each compartment (capsule) remains permeable to small molecules such as nutrients from growth media (but remains impermeable to nanoscale entities such as enzymes or nanoparticles). Secondly, the experiment illustrates that cross-talk between different microbial species can be studied using MCCs. To our knowledge, this is the first example of a *cellular* cascade process within an MCC or artificial-cell construct. In the future, we believe that MCCs are likely to be applicable in a variety of contexts. In terms of physical and chemical studies, we envision aqueous catalytic processes involving distinct catalysts (*e.g.*, nanoparticles) sequestered in different compartments. In terms of biological studies, MCCs could be used to explore the co-culture of competitive species and the cross-talk between one kingdom of microorganisms to another.^42^

## Materials and methods

### Materials and chemicals

The following chemicals were obtained from Sigma-Aldrich: the biopolymers, sodium alginate (from brown algae, medium viscosity) and chitosan oligosaccharide lactate (5000 Da, degree of deacetylation > 90%); the nonionic surfactant, Pluronic F127; and the inorganic salt, calcium chloride dihydrate. PBS and LB broth were obtained from Life Technologies. Magnetic nanoparticles (EMG 304) with a nominal diameter of 10 nm were obtained as an aqueous dispersion (4.5 vol% particles) from Ferrotec. Fluorescently-labeled green and red microparticles (0.7–0.9 μm diameter) were purchased from Spherotech as an aqueous dispersion (1% w/v of particles).

### Device fabrication

The microfluidic device described in Fig. 2 was fabricated as follows (a photo showing the different components is provided in Fig. S1†). A seven-barrel glass capillary (1.5 cm long) from World Precision Instruments (WPI) was inserted into the male of a Luer adapter tee (Cole-Parmer, EW-45508-85). A 5 cm-long square capillary from Vitrocom (8320, with a 200 μm ID) was then inserted into the center of the seven-barrel capillary, and the whole setup was sealed by an epoxy adhesive (Devcon 5-min epoxy). Another glass capillary from Vitrocom (CV0508, with a 50 μm ID) was hydrophobically modified according to previously published methods.^46^ This capillary was inserted into a flexible capillary (Polymicro, TSP100200, polyimide-coated, and with a 100 μm ID) and sealed by epoxy. This flexible capillary was then threaded through the square capillary on one end and on the other end through a male Luer syringe connector with 1/16 in. hose barb (Cole Parmer, EW-45505-00). The extruded piece of the flexible capillary on the side of the barb was then inserted and epoxied into a piece of Tygon tubing (Cole Parmer, EW-06509-13). A P1000 plastic pipette was cut to encase around the capillary apparatus to focus the gas stream, then sealed with epoxy. Note that the nesting of multiple capillaries over a range of sizes was done to ensure that the smallest capillary (50 μm) was centered within the device, so that the gas flowed uniformly around its tip. It is through the tip of this smallest capillary that the liquid droplets emerged. Also, the nesting eliminated any vibration of this capillary due to the gas flow.

The device to fabricate the MCCs was considerably simpler. Instead of the four capillaries above, only two were required for this case. The seven-barrel glass capillary was used again. A circular capillary from Vitrocom (CV2033 with a 200 μm ID) was hydrophobically modified as per previously published methods.^46^ This was inserted into the center of the seven-barrel capillary. One end of the above circular capillary was then directly threaded into the male Luer syringe connector with 1/16 in. hose barb. Tygon tubing was then capped over the barb, and the entire setup was sealed by epoxy.

In addition to the above capillary device, our setup (see Fig. 2 and S1†) consisted of an adjustable syringe pump for the liquid feed (NE-1002X, purchased from ; http://syringepump.com), a gas flow-regulator (from Techon Systems), a function generator (from BK Precision), and a cylinder of compressed air or nitrogen (from AirGas). The settings for gas flow were set at timed pulses (P4) over 0.1 s, with consecutive pulses separated by the pulsing frequency *f* (see schematic in Fig. S1c†). The pressure of the gas was set at a constant value of 14 psi. The gas output was connected to the other end of the Tygon tubing from the capillary device. A disposable syringe was connected to the Tygon tubing through a Luer lock. A piece of paper towel wetted with water was folded into the syringe to humidify the gas stream entering the device.

### Synthesis of inner capsules and MCCs

For bare capsules, the feed solution consisted of 2.25 wt% alginate dissolved in PBS and it was filtered through 0.45 μm cellulose syringe filters (from Millipore) prior to use. As noted in Fig. 3, the feed flow rate was varied between 0.25 to 1 μL min^–1^ while the pulsing frequency of the gas was varied between 1 to 7 Hz. Droplets were introduced into a reservoir solution consisting of 1 wt% chitosan, 1 wt% CaCl_2_ and 0.3 wt% Pluronic F127. The reservoir was held on an adjustable stage (see photo in Fig. S1d†) so that the vertical distance between the capillary tip and the reservoir could be varied (typically, this distance was maintained at about 2 in.). Once the droplets enter the reservoir, they were incubated for a period of about 30 min, whereupon they were converted to capsules. The presence of the Pluronic surfactant in the reservoir solution ensured that the droplets became immersed in the solution rather than collecting on the liquid surface. After formation, the capsules were washed three times with PBS and then resuspended in PBS.

For preparing the magnetic capsules, the feed consisted of alginate with the EMG 304 nanoparticles. To prepare this feed, 1.5 g of 3 wt% alginate solution was combined with 0.5 g of the EMG 304 dispersion diluted 10× with PBS (final alginate concentration was 2.25 wt% as before; final concentration of the magnetic particles was 0.05 wt%). Similarly, for preparing fluorescent capsules, 1.8 g of 2.5 wt% alginate was combined with 0.2 g of the dispersion of fluorescent microparticles (red or green). For preparing capsules containing bacterial cells, 1.5 g of 3 wt% alginate solution was combined with 0.5 g of the cell pellet.

For preparing MCCs, the capsules were resuspended in a 2 wt% alginate solution, and this suspension was used as the feed. The number density of capsules in this suspension was varied from 1000 to 10 000 capsules per mL. The feed flow rate in this case was between 10 to 60 μL min^–1^ while the pulsing frequency of the gas was again between 1 to 7 Hz. Droplets bearing capsules were introduced into a reservoir with identical composition as above. Following an incubation time of 30 min, the resulting MCCs were washed three times with PBS and then resuspended in PBS.

### Image analysis

Bright-field and fluorescence microscopy on the capsules and MCCs was performed using an Olympus MVX10 MacroView fluorescence stereomicroscope equipped with a DP72 Camera. Images were taken with red and green filter sets as well as in brightfield mode, and these were overlaid using Adobe Photoshop to visualize both colors simultaneously.

### Cell culture

Two types of *E. coli* reporter strains were used: W3110 (Δ*luxS*) + pCT6 + pET-dsRed for red fluorescent expression and W3110 (Δ*luxS*, Δ*lsrFG*) + pCT6 + pET-GFPuv for green fluorescent expression. BL21 (LuxS+) was used as AI-2 producers, and W3110 (Δ*luxS*, Δ*lsrFG*) + pCT6 + pET-Venus was used as reporters of AI-2. Plasmid constructs are described by Tsao *et al.*^41^ All *E. coli* strains were grown in LB medium at 37 °C and 250 rpm until an optical density (at 600 nm) of 0.4 was reached (for the Venus-producing strain alone, the medium was supplemented with kanamycin and ampicillin at 50 μg mL^–1^ per antibiotic). Subsequently, cultures were centrifuged at 3900 rpm for 7 min and resuspended in 0.5 g of PBS. Once encapsulated, the capsules were shaken at 37 °C to observe the bacterial responses.

## Conflicts of interest

There are no conflicts to declare.

## Supplementary Material

Supplementary information
