# Supplementary material for: A new design for an artificial cell: polymer microcapsules with addressable inner compartments that can harbor biomolecules, colloids or microbial species
Source: Chem Sci. 2017 Aug 17;8(10):6893–903. doi: 10.1039/c7sc01335c (PMC6103254; doi:10.1039/c7sc01335c)
Supplement: Supplementary file 1 [file SC-008-C7SC01335C-s001.pdf]

Electronic Supplementary Material (ESI) for Chemical Science.
This journal is © The Royal Society of Chemistry 2017

<u>Supporting Information for:</u>

A New Design for an Artificial Cell: Polymer Microcapsules with Addressable Inner Compartments that Can Harbor Biomolecules, Colloids or Microbial Species

Annie Xi Lu,[1†] Hyuntaek Oh,[1†] Jessica L. Terrell,[2] William E. Bentley,[1,2] and Srinivasa R. Raghavan[1,2]*
[1]Department of Chemical and Biomolecular Engineering, University of Maryland, College Park, MD 20742, USA
[2]Fischell Department of Bioengineering, University of Maryland, College Park, MD 20742, USA
[†]Equal contribution; *Corresponding author. Email: sraghava@umd.edu

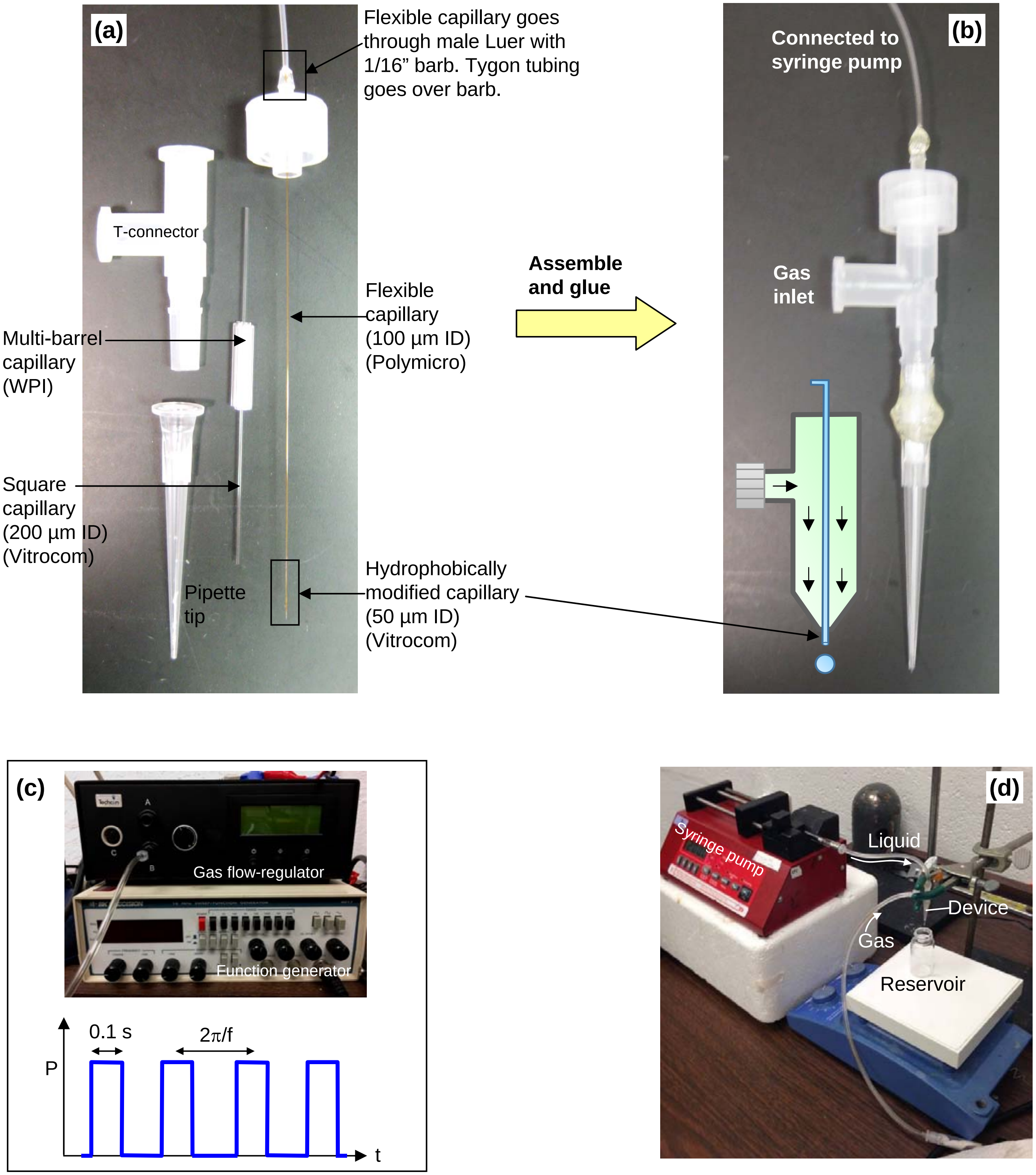

Figure S1. Photos and details of the microfluidic setup used to generate capsules and MCCs. The components of the microfluidic device are shown in (a). After assembly and gluing with epoxy, the final device is shown in (b). The schematic in Figure 2 is reproduced as an inset to show the correspondence to the actual device. Note that the liquid finally emerges out of the capillary with a 50 µm ID. In (c), a photo of the function generator and gas flow-regulator is shown. The gas is sent as pulses at a pressure *P*, with each pulse over a duration of 0.1 s and with the spacing between pulses dictated by the frequency *f*. In (d), a photo of the device in operation is shown, along with the syringe pump and collection reservoir (vial).

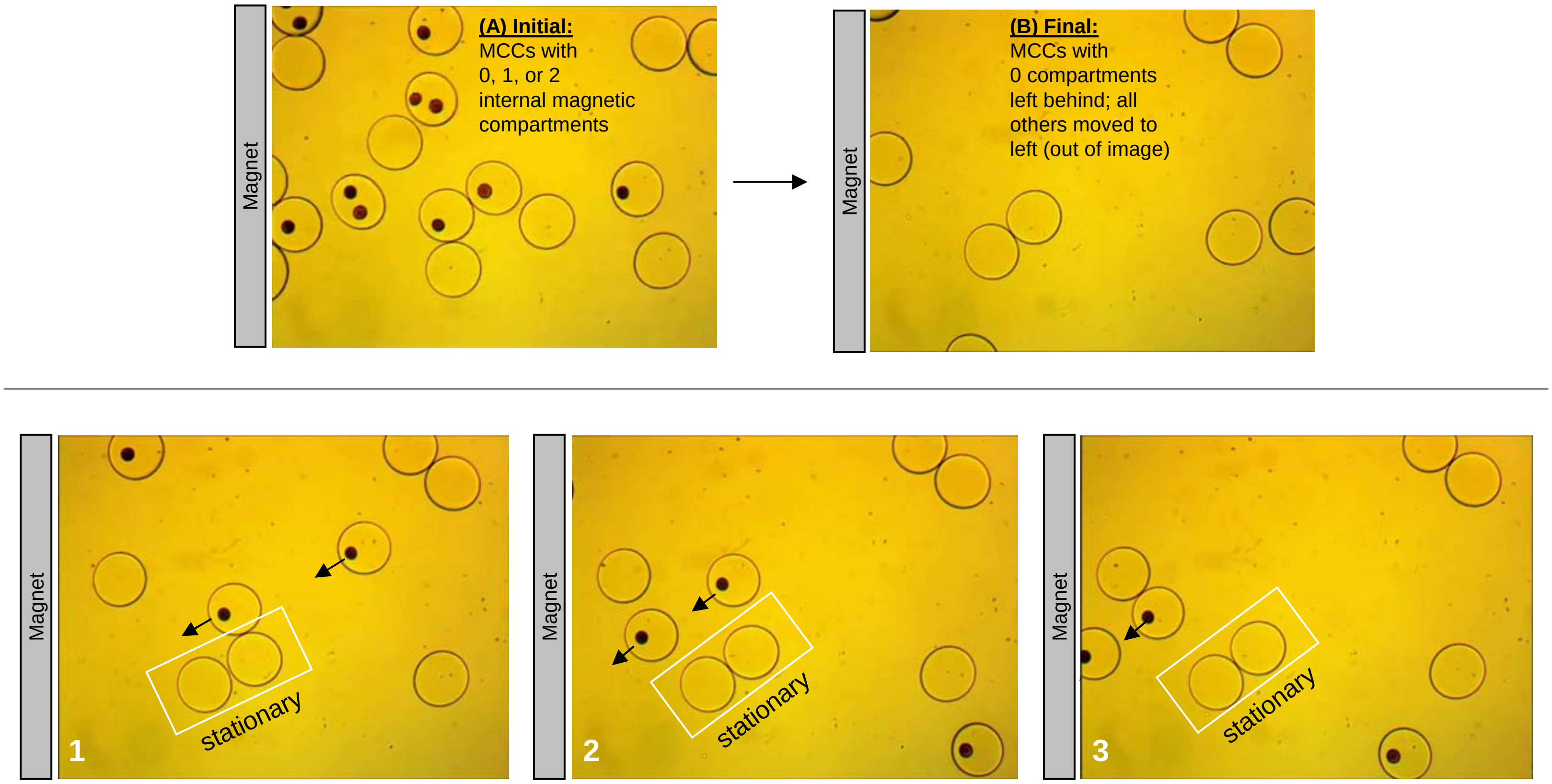

Figure S2. Magnetic sorting of multicompartment capsules (MCCs). The images in this figure are stills from a movie. (Top panel) The initial and final images from the movie. In the initial image, a population of MCCs is shown in which the internal compartments (smaller capsules) have magnetic nanoparticles and hence a brown color. Some of the MCCs do not have an internal compartment (i.e., they are not really MCCs) while others have 1 or 2 such compartments. At t = 0, a bar magnet is placed to the left of this volume. This induces the MCCs with 1 or 2 internal compartments to move towards the left (out of the screen) due to their magnetic properties. In the final image, the bare MCCs alone are left behind. (Bottom panel) A succession of stills from the movie are shown in Images 1, 2, and 3 to demonstrate that the MCCs with arrows are moving towards the left relative to the stationary pair of MCCs marked with a box.
